# A Comprehensive Review of the Contemporary Methods for Enhancing Osseointegration and the Antimicrobial Properties of Titanium Dental Implants

**DOI:** 10.7759/cureus.68720

**Published:** 2024-09-05

**Authors:** Fahad A Bakitian

**Affiliations:** 1 Department of Restorative Dentistry, Faculty of Dental Medicine, Umm Al-Qura University, Makkah, SAU

**Keywords:** implant surface modification, titanium, peri-implantitis, implant osseointegration, dental implant

## Abstract

Titanium dental implants with various restorative options are popular for replacing missing teeth due to their comfortable fit, excellent stability, natural appearance, and impressive track record in clinical settings. However, challenges such as potential issues with osseointegration, peri-implant bone loss, and peri-implantitis might lead to implant failure, causing concern for patients and dental staff. Surface modification has the potential to significantly enhance the success rate of titanium implants and meet the needs of clinical applications. This involves the application of various physical, chemical, and bioactive coatings, as well as adjustments to implant surface topography, offering significant potential for enhancing implant outcomes in terms of osseointegration and antimicrobial properties. Many surface modification methods have been employed to improve titanium implants, showcasing the diversity of approaches in this field including sandblasting, acid etching, plasma spraying, plasma immersion ion implantation, physical vapor deposition, electrophoretic deposition, electrochemical deposition, anodization, microarc oxidation, laser treatments, sol-gel method, layer-by-layer self-assembly technology, and the adsorption of biomolecules. This article provides a comprehensive overview of the surface modification methods for titanium implants to address issues with insufficient osseointegration and implant-related infections. It encompasses the physical, chemical, and biological aspects of these methods to provide researchers and dental professionals with a robust resource to aid them in their study and practical use of dental implant materials, ensuring they are thoroughly knowledgeable and well-prepared for their endeavors.

## Introduction and background

Recent advancements in dental implant materials and technologies have made implants the preferred choice for patients with missing teeth [1]. Implant-supported prostheses provide highly effective solutions to restore the oral function and aesthetics of partially and fully edentulous patients, with excellent reported survival rates reaching 94.6%, instilling confidence in their reliability [2]. Commercially pure titanium (cp Ti) and titanium alloy (Ti-6Al-4V) are among the most common dental implant biomaterials widely used since the 1960s. Their popularity is due to their excellent biocompatible properties, unique mechanical and chemical properties, and good corrosion resistance [3].

Osseointegration is a vital and complex process that plays a crucial role in the successful integration of titanium (Ti) implants within living bone [4]. This integration provides stability and ensures the long-term effectiveness and durability of the implants in clinical applications [4,5]. Osseointegration begins with a direct connection between the alveolar bone and the surface of the Ti implant without any intervening connective tissue, followed by natural fixation through ongoing bone apposition and remodeling around the implant. The area where the implant and tissue meet is a highly dynamic and active interaction site [4,5]. This interaction involves issues related to the biocompatibility of biomaterials with the body and changes in the mechanical environment surrounding the implant.

Peri-implantitis is a common challenge in dental practice [6,7]. It is characterized by a destructive inflammatory process that affects the tissues surrounding dental implants, ultimately leading to implant failure [6,7]. The prevalence of peri-implantitis can vary significantly, affecting up to 22% of patients and 25% of implants that have been in place for at least five years [7]. The condition begins with peri-implant mucositis, which is inflammation of the soft tissue around the implant and, if left untreated, progresses to the loss of alveolar bone surrounding the implant. Inadequate oral hygiene is the primary factor contributing to the progression of peri-implantitis, as it can result in the accumulation of bacterial plaque [6,7]. It is crucial to recognize that dental implants employed in clinical practice have limited antimicrobial properties. Consequently, bacterial colonization and biofilms on implant surfaces can lead to the development of peri-implantitis [8], highlighting the importance of effective prevention strategies rather than solely relying on treatment.

Over the last 30 years, extensive research has focused on enhancing Ti implants' surface characteristics to improve osseointegration and reduce the risk of peri-implantitis [9-11]. Various methods have been employed to modify the surface of implants, creating complex structures at different scales and significantly enhancing biofunctionalization [9-11]. These advancements have led to significant progress in implant dentistry. Therefore, this comprehensive review offers a valuable compilation of surface modifications to enhance Ti implants' osseointegration and antimicrobial capabilities, serving as a solid reference for further research and practical application in this field.

## Review

**Figure 1:**
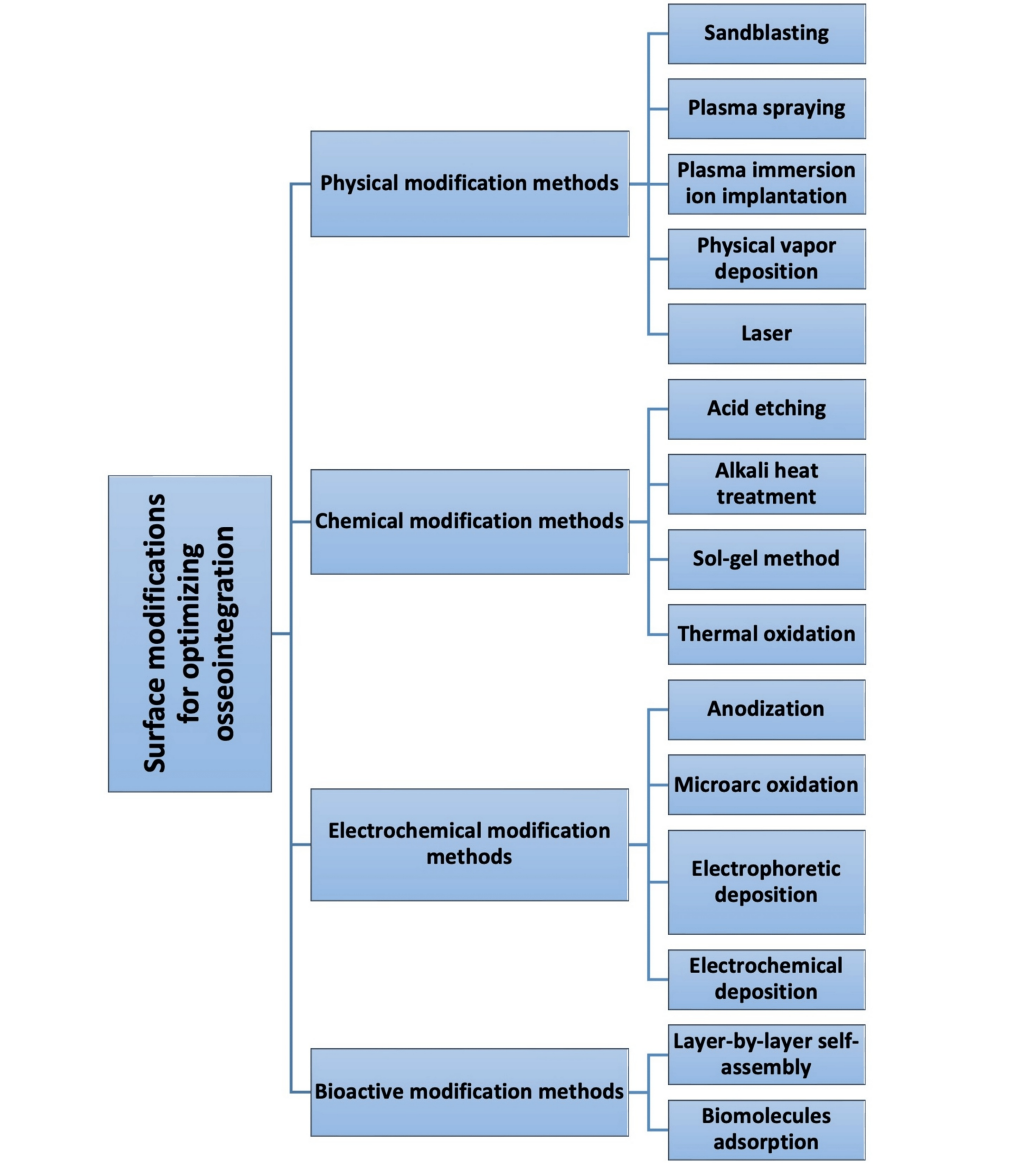
Surface modification methods used for enhancing osseointegration

Given the recent surge in popularity of implants as a treatment option, the importance of surface modifications to enhance implant integration with surrounding tissues and promote osseointegration is on the rise. Various methods have been used to modify implant surfaces (Figure 1), each with pros and cons. Moreover, recent research has highlighted a critical need to improve the antimicrobial properties of implant surfaces to prevent peri-implant infections. The following sections will thoroughly discuss the surface modification characteristics of Ti implants from physical, chemical, and biological perspectives.

Physical and chemical surface modifications for optimizing osseointegration

Sandblasting and Acid Etching

Sandblasting involves using a high-speed jet beam created by compressed air to spray materials of different particle sizes onto the implant surface, changing the surface roughness [12]. Using this subtractive approach increases the surface area of implants, promoting cell adhesion and improving the osseointegration capacity [12]. TiOblast^®^ (Astra Tech, Mölndal, Sweden) is an example of a commercial implant brand utilizing this subtractive surface modification method. Previous studies have shown that adjusting the sandblasting particle size and injection rate can alter the roughness of the Ti implant surface [13,14]. Sandblasting can be performed with or without an acid etching treatment. When combined, sandblasting with alumina particles of large grit (0.25-0.50 µm) is first performed to create a primary rough structure of 10~30 µm [15,16], followed by acid etching with hydrogen chloride/sulfuric acid or their mixtures at high temperatures to obtain a secondary rough structure with micropores of 1 to 3 µm on the Ti implant surface. The primary rough structure promotes osteoblast adhesion, while the secondary rough structure facilitates osteoblast proliferation and differentiation. The surface topography of this multi-level rough structure is inductive to osseointegration [15,16]. SLA Straumann^®^ is a widely used commercial dental implant manufactured by Straumann (Straumann Institute, Basel, Switzerland). It is known for its surface modification, which involves a combination of sandblasting with alumina and acid etching processes. Previous studies consistently indicate that sandblasting with acid etching outperforms plasma spraying of Ti surfaces regarding bone response during osseointegrations under unloaded and loaded implant conditions [17]. Additionally, comparable bone-implant contact (BIC) values were found between sandblasting with acid etching and hydroxyapatite (HA) coating on implant surfaces, with the latter showing more evidence of resorption [17].

Alkali Heat Treatment

Alkali heat treatment is used to enhance the surface chemistry of implants, making them more biologically active and improving their interaction with surrounding tissues [18,19]. This process involves immersing Ti implants in a strong alkali solution of specific concentration, followed by heat treatment at 300-800°C to create a porous oxide layer. This chemical treatment significantly increases the surface roughness of Ti and develops micron-sized porous structures, promoting HA deposition [18,19]. Moreover, pre-treating the Ti surfaces with acid before alkali heat treatment further increases surface roughness and enhances the rapid deposition of HA compared to alkali heat treatment alone, resulting in stronger bonding between the deposited HA and the Ti substrate [20].

Plasma Spraying

Plasma spraying, a thermal process that utilizes an electrical-driven arc to create high-temperature ionized gas, is a crucial technique in biomaterials and dental implants [21,22]. It melts materials into a molten or semi-molten state and sprays them onto the pretreated implant surface at high speed. ITI-TPS^®^ (Straumann Institute, Waldenburg, Germany) represents a commercial example of implants utilizing this surface modification method. This physical, additive surface modification method offers advantages such as rapid deposition, thick coatings, and low cost, making it an essential study area in biomaterials research. The HA coating commonly used in clinical practice is formed by spraying HA particles on the implant surface at high temperatures and then rapidly cooling it [21,22]. HA coatings can improve osseointegration and promote rapid bone repair after implantation, but plasma spraying is associated with challenges like phase impurities and poor adhesive strength between the HA coating and Ti surface [23-25]. Additionally, differences in thermal expansion coefficients between the HA coating and the Ti surface during spraying can lead to high residual stresses, risking coating delamination and degradation, thus hindering long-term implantation [23,25]. 

Although HA is a well-known osteoconductive biomaterial, its effectiveness as an implant coating may be limited due to its lack of specific bone-forming properties [24,25]. Therefore, researchers have modified HA coatings by incorporating different materials such as organic bioactive agents, like vascular endothelial growth factors, and inorganic molecules, like magnesium (Mg) and silicon (Si) to encourage bone growth [23,26]. These modifications improve and speed up the osseointegration process. Additionally, the modified coatings have led to a more robust bond between the bone and implant than pure HA, indicating that the doped HA coating significantly enhances osseointegration [23,25].

Plasma spraying also uses a plasma flame to melt Ti powder, which is then sprayed onto the implant surface to create a porous titanium dioxide (TiO_2_) coating that encourages osseointegration with long-term stability [27,28]. The porous layer provides an ideal topography for bone cells to adhere to and grow, promoting bone growth and implant anchorage without compromising the implant's structural integrity [27]. Bioactive elements like Mg and strontium (Sr) ions are added to the TiO_2_ coating to enhance osteoinduction and osteoconduction [28]. However, it is important to note that this process has limitations in controlling the uniform coating thickness, which is crucial for optimal implant performance [27,28].

Plasma Immersion Ion Implantation (PIII)

PIII is another physical surface modification that can improve the osseointegration capability of Ti implants [29]. This method involves injecting an ion beam into the implant surface, where the ions interact physically and chemically with the atoms or molecules on the implant surface. As the ions lose energy, they ultimately reside in the implant surface, leading to changes in the surface composition. Previous studies have used PIII to introduce silver (Ag) nanoparticles onto Ti surfaces to improve the proliferation of osteoblast-like cells while effectively inhibiting the growth of *Staphylococcus aureus* (*S. aureus*) and *Escherichia* *coli* (*E. coli*) [29,30]. However, the results show that the corrosion resistance of Ti samples is slightly reduced by the introduction of Ag [29,30].

Physical Vapor Deposition (PVD)

PVD is a versatile method for applying thin-film coatings in a vacuum, facilitating the production of coatings comprising pure metals, metal alloys, and ceramics, typically ranging from 1 to 10 µm in thickness [31,32]. The process encompasses several stages, during which the material changes from a solid to a vapor phase, subsequently recondensing into a thin film on the substrate. Two primary processes are commonly used in PVD, the first is sputtering which involves bombarding the material with a plasma discharge causing it to vaporize, while the second is evaporation, where the material is heated until it vaporizes and then condenses onto the substrate.

Magnetron sputtering is a widely used PVD method that is highly efficient in applying versatile coatings on Ti implant materials [33,34]. This method is particularly advantageous for materials with high melting points which are unsuitable for evaporation. Magnetron sputtering has been employed to produce dense and uniform coatings of HA or biphasic ceramics such as HA and calcium phosphate (CaP) on Ti substrates, ensuring strong adhesion to the substrate [33,34]. The created HA-coated implants demonstrated enhanced bioactivity, as indicated by the development of a porous and interconnected HA layer that closely mimics natural bone [33,34]. This surface modification can also improve BIC values with favorable hemocompatibility [33,34].

Laser Treatments

Laser technology plays a crucial role in two essential applications of dental implantology, coating and texturing. In the coating application, precise laser pulses evaporate the target materials which condense on the substrate to create a thin, protective coating [35]. In the texturing application, the laser prepares specific surface topographies on the implant, resulting in a textured surface that can significantly improve osseointegration [36]. Laser-Lok® (BioHorizons, Birmingham, Alabama) is an example of a commercial dental implant that utilizes laser technology to modify the implant.

Pulsed-laser deposition (PLD) is a laser treatment used to create thin-film coatings on implant surfaces, offering exceptional versatility [35,37]. In this method, laser pulses eject material from a particular object, usually a solid, generating a plasma. The ejected material subsequently deposits onto a substrate, forming a thin layer. PLD-produced HA coatings demonstrate remarkable bonding strength with Ti substrates and higher purity than plasma-sprayed coatings [37]. Furthermore, HA coatings generated through PLD promote osteoblast differentiation and possess strong osteogenic properties owing to their granular surface and superior crystallinity [38].

Selective laser melting (SLM) utilizes metal powder that is melted by a laser beam and is solidified into a mold. The rough microtopography of SLM creates nanostructures on Ti surfaces, establishing a hierarchical micro-nano topography that effectively improves osseointegration [36,39]. Previous studies applied SLM to manufacture Ti6Al4V implants with microstructured surfaces and then further nanostructured them by electrochemical anodization to form TiO_2_ nanotubes [36]. They were then bioactivated by HA to enhance their osteogenic properties, indicating that this modification could promote cell maturation and surface mineralization on the implant surface [39].

Thermal Atmospheric Oxidation

Thermal atmospheric oxidation is a chemical surface modification method to modify the Ti surfaces [40]. It refers to the oxidation that occurs when metals are exposed to high temperatures in the atmosphere in the presence of oxygen (O_2_). The process occurs without an external electric field, forming an oxide layer on the implant surface, significantly improving the contact between osteoblasts and the implant to speed up osseointegration [40,41]. The produced oxide layer enhances the bone-forming properties of the implant surface by improving wettability [40,41]. However, the surface composition of the Ti implants can vary depending on the different oxidizing atmosphere treatments they are exposed to, resulting in deposits comprising various phases [42]. For instance, the oxidation of Ti in air results in the formation of the rutile bioactive phase of TiO_2_ [42]. Lower O_2_ levels in the air promote increased O_2_ diffusion across the Ti surface, initiating a gradual and sufficient Ti oxidation. Conversely, when Ti is exposed to pure O_2_, it forms titanium monoxide (TiO) and TiO_2_ [40]. The high O_2_ concentration causes rapid oxidation, creating an oxide layer on the surface that prevents further oxidation. Air-treated surfaces exhibit significantly greater hydrophilicity, HA formation, cell attachment, and proliferation than surfaces treated with pure O_2_ [42].

Sol-Gel Method

The sol-gel method is highly effective for creating Ti implant coatings, such as HA and TiO_2_ [43,44]. This chemical surface modification is characterized by its low processing temperatures, cost-effectiveness, and the ability to produce consistent and pure coatings. The process involves immersing the substrate in a sol-gel precursor solution and repeatedly withdrawing and heating it to create a bioactive surface layer. Sol-gel films are often made through dip-coating, spin-coating, and spraying techniques [43,44]. However, an alternative electrochemical deposition method has gained prominence recently as an innovative approach for creating sol-gel films [45].

HA coatings derived from the sol-gel method demonstrate superior corrosion resistance, strong adhesion to the substrate, enhanced bioactivity, and improved morphological and structural characteristics [43-45]. However, cracks are often observed in coatings obtained by this method [46]. Moreover, the impact of thermal effects needs to be considered, so its current clinical use is subject to certain limitations.

Electrochemical surface modifications for optimizing osseointegration

Anodization

Anodization or anodic oxidation is a technique used to modify the surface of metals through oxidation. This process involves the creation of an oxide film on a metal surface by electrochemical means, resulting in a microstructured surface with micrometer-sized pores on Ti substrates [47,48]. During anodization, positive and negative ions in the electrolyte move toward the cathode and anode under an electric field, leading to an oxidation-reduction reaction. The latter causes the formation and disappearance of the oxide film on the Ti surface, creating uniform micro-nano pores or small tubes. The oxide coating produced by anodization can alter the surface color, corrosion resistance, hardness, and other properties of Ti-based materials [47,48]. Moreover, anodization can create different surface structures such as nanotubes, nanopores, and micro-nano textures on Ti-based materials by using various electrolytes and adjusting the processing parameters [49-52]. A popular example of a commercial implant using this surface modification method is the TiUnite® brand (Nobel Biocare, Gothenburg, Sweden).

Ti implants naturally develop a biologically inactive layer of TiO_2_ on their surface, but anodization leads to the formation of bioactive TiO_2_, specifically rutile, and anatase, on the implant surface [47,48]. Previous studies have shown that immersing Ti in a sulphuric acid electrolyte solution for anodizing treatment can lead to the formation of anatase, and increasing the current density or decreasing the electrolyte concentration can further increase the proportion of anatase produced on the Ti surface [47,48].

Incorporating biofunctional elements such as calcium (Ca), phosphorus (P), copper (Cu), and Ag into the electrolyte has been tested to modify the coating properties. A biomimetic CaP in anodized coating with a ratio similar to natural HA can promote BIC values [53,54]. Elements such as Sr and Mg have been incorporated to encourage osseointegration [53,54]. Additionally, some studies have used Cu in anodized coatings during hydrothermal treatment to produce surfaces that up-regulate the expression of angiogenesis-related factors and osteogenesis-related genes in cultured rat bone marrow stem cells [55].

The preparation of TiO_2_ nanotubes by anodization has received widespread attention because of their promising biocompatibility and the ability to enhance osteoblast adhesion and proliferation [49-52]. TiO_2_ nanotubes have a hollow tubular structure which significantly improves the biological activity of Ti due to their large specific surface area and strong adsorption capacity. Moreover, incorporating TiO_2_ nanotubes on polished Ti surfaces significantly increased the surface roughness and enhanced wettability [50]. This rougher and more hydrophilic surface provides more anchorage sites for cellular contacts, thus considerably improving osteoblast adhesion and proliferation. Besides their direct influence on cell behavior, TiO_2_ nanotubes fabricated on Ti-based implant materials serve as carriers for bioactive molecules [51,52]. These structures can regulate the release of active molecules, enhancing osseointegration [52], indicating the potential for these nanotubes to revolutionize the field of biomedical engineering.

Microarc Oxidation (MAO)

MAO is an improved version of anodic oxidation technology. This innovative electrochemical surface modification process can create bioactive TiO-based coatings on Ti substrates [54,56]. In this process, the implant is placed in an electrolyte solution and subjected to high voltage forming small, localized discharges. Various factors, such as the composition of the electrolyte, voltage, and current, can influence the MAO coating properties [54,56]. The Ti surface develops an oxide film with a thickness of tens of microns, a dense inner layer, and a porous outer layer. The micro-nano bioactive TiO_2_ coatings produced by MAO modification can enhance cell adhesion on the implant surface [56]. The MAO coating has been improved by incorporating zinc (Zn), Ca, and P elements, increasing the binding strength and demonstrating an excellent ability to induce HA deposition while exhibiting good antimicrobial properties [57]. Moreover, using a tetraborate electrolyte creates TiO-based coatings with a dual-scale porous structure on Ti surfaces [57]. The coating combined micro and nanopores, thus super hydrophilicity to significantly improve the adhesion, proliferation, and differentiation of human bone marrow mesenchymal stem cells, leading to an excellent osseointegration effect and an increased implantation success rate [57]. In another study, a porous TiO_2_ coating was prepared on ultrafine-grained Ti using MAO technology in an electrolyte containing Ca, P, and Si, which exhibited high surface energy and surface roughness to enhance osteoblast adhesion and diffusion [58].

Electrophoretic Deposition

Electrophoretic deposition involves applying high voltage to a conductive substrate to cause charged particles from the suspension to adhere to the implant [59,60]. This method enables the application of ceramic coatings on intricate-shaped implants. Additionally, electrophoretic deposition offers precise regulation over coating characteristics such as thickness and composition, in contrast to traditional methods like plasma spraying [59,60]. However, using this deposition method, the densification of ceramic coatings at high sintering temperatures can lead to the deterioration of the metal substrate and decomposition of the ceramic coating. Thus, it is recommended that the sintering temperature be kept below 1000°C to minimize these adverse effects, producing dense and stable coatings on metallic implants using electrophoretic deposition [61]. One method to reduce the sintering temperature is using nanosized ceramic powders with a high specific surface area [61]. Previous studies have used different wet processes to electrophoretically deposit HA nanoparticles onto the Ti substrate, creating HA nanoparticle coatings with various regular and irregular shapes [61,62]. The structure and shape of the HA nanoparticles are crucial for the coating quality, especially in preventing cracking. It has been shown that regular-shaped nanoparticles are less likely to cause coating cracking [62].

Electrochemical Deposition

Electrochemical deposition is a commonly used and cost-effective method to coat Ti implant surfaces with an HA layer [63,64]. This technique results in a stable, reliable, and controllable coating crystallinity [63,64]. HA coatings applied through electrochemical deposition significantly improve bioactivity and corrosion resistance [65]. Combined with metal oxide nanocomposites, these coatings can enhance mechanical strength and improve corrosion protection [66]. The process typically involves depositing HA nanoparticles onto Ti substrates using a mixture of Ca and P ions. Previous research has demonstrated that adjusting the applied current and its duration in the electrochemical deposition process allows for effective control over the thickness of the HA layer [65,66]. Instead of a dense HA layer, a porous interconnected network of the HA layer is deposited on the implant surface, enhancing bioactivity and increasing the biomineralization capacity of electrochemically coated Ti implants.

Bioactive surface modification for optimizing osseointegration

Layer-by-Layer Self-Assembly (LBL)

The LBL method involves creating multilayer films through interactions between oppositely charged polyelectrolytes [67]. This method offers new possibilities for modifying Ti implant surfaces with several advantages including promoting osteogenesis and osseointegration and introducing drugs onto the surface of implants for bioactivation [67,68]. The LBL method forms multilayer films on the smooth Ti surface using sodium hyaluronate and chitosan/small interfering RNA nanoparticles as polyanions and polycations [68]. These modified Ti surfaces significantly promote osteogenesis and cell differentiation [68]. Moreover, multilayers of peptides have been developed using this method by exposing precharged poly(lactic-co-glycolic acid) (PLGA) and nano HA membranes to polyelectrolytes [67,69]. This process results in a multilayer gradient of peptide layers on the implant surface which significantly improves cell attachment and growth, directing the differentiation of mesenchymal stem cells, and promoting mineralization.

Biomolecule Adsorption

Biomolecules, such as proteins, are essential compounds produced by living organisms, playing vital roles in various biological processes including osseointegration [70]. Biomolecule adsorption occurs when molecules or ions in the surrounding medium attach to the implant surface through intermolecular forces or chemical bonds. For instance, when biomedical implants come into contact with biological fluids such as blood plasma and saliva, extracellular matrix protein adsorption to implant surfaces creates an immediate biological coating. These adsorbed proteins set the stage for subsequent interactions with host cells, influencing the success of biomedical implants [70-72].

Previous studies have shown that nonpolar, high surface tension, and electrically charged substrates are generally preferred for protein-based coatings [71,72]. To achieve successful protein-related coating, the implant surface typically undergoes various treatments. For instance, porous Ti implants have been prepared with super hydrophilic and negatively charged surfaces through alkaline heat treatment, adsorbing positively charged protamine coating [72]. Biofunctionalization of the basal layer was achieved by further immobilizing exogenous bone morphogenetic protein-2 on the coating surface [72]. The adsorbed protamine coatings effectively inhibited the initial burst release of the adsorbed protein and achieved uniform protein distribution and sustained biomolecule release. Compared with untreated Ti, the adsorbed protamine coating showed good cytocompatibility at the initial stage and promoted cell adhesion [72]. The findings suggest that combining inorganic and organic surface modifications can increase the osseointegration potential of implant materials.

Several studies have investigated other protein-based coatings on implant surfaces [73,74]. In one study, researchers covalently linked collagen to Ti implants treated with vapor-salinization to achieve a high concentration of amine groups on the surface [73]. This integration of collagen led to improved viability and attachment of mesenchymal stem cells on the implant surface. In another study, the attachment of an elastin-like protein with an extended arginyl glycyl aspartic acid (RGD) sequence to the Ti surface increased the transformation of mesenchymal stem cells into bone cells and enhanced bone mineralization, thereby improving osseointegration capacity [74]. Moreover, RGD facilitates the attachment and proliferation of bone-related cells, leading to improved osseointegration [74]. Adsorption of different biomolecules, such as chitosan, sodium alginate, and pectin, has also been investigated as bioactive coatings on the Ti surface, with favorable effects on the proliferation and differentiation of osteoblasts [75].

Surface modifications for optimizing antimicrobial properties

**Figure 2:**
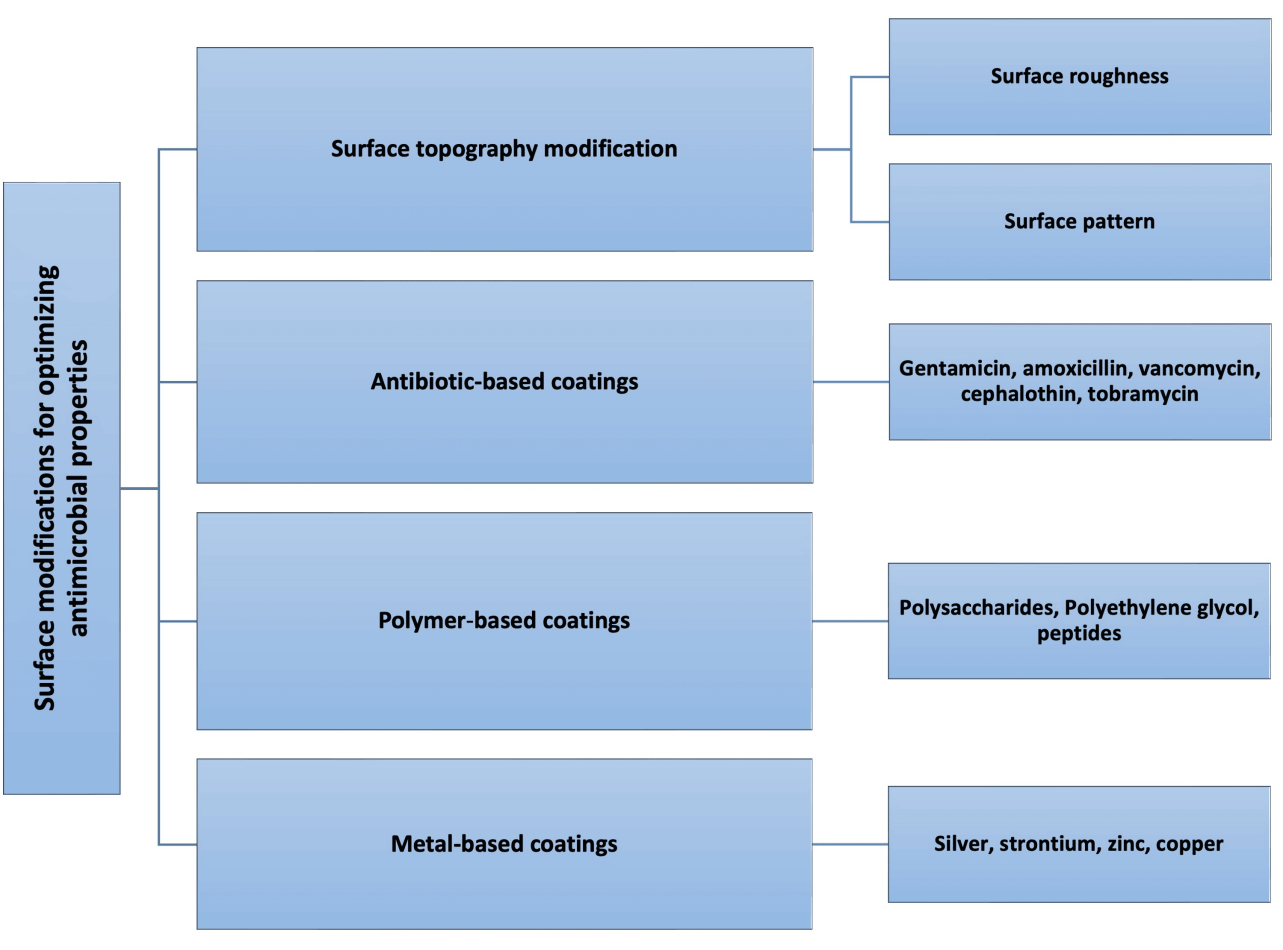
Surface modification methods used for enhancing antimicrobial properties

Several surface modification methods have been used to enhance the antimicrobial properties of Ti surfaces (Figure 2). The physical modification of the implant surface includes its topography, that is, the surface roughness and profile shape, which significantly influence bacterial adhesion and biofilm formation [76]. Additionally, implant surfaces were chemically and electrochemically modified using multifunctional coatings containing antimicrobial agents such as antibiotics, polymers, peptides, polysaccharides, and metal ions.

Surface Topography Modification

Several studies were performed to understand the surface topography that hinders bacterial colonization and biofilm formation [76-80]. Implant surface roughness is usually obtained through sandblasting, acid etching, and anodic oxidation to improve the adhesion and proliferation of osteoblasts and fibroblasts [77]. However, when the surface is made rougher, it provides more attachment sites, increasing bacterial adhesion [78]. Conversely, smooth surfaces resist bacterial adhesion and biofilm formation. However, some studies on the impact of surface roughness on the adhesion and growth of fibroblasts and bacteria on Ti surfaces have yielded conflicting results showing that smooth surfaces are more conducive to fibroblast and bacterial adhesion and growth than rougher surfaces [79]. Those inconsistent findings could be related to variations in the materials and bacterial strains employed in the studies. The concept of nanoroughness has been suggested as an effective means of preventing the adhesion of microorganisms [80]. Surfaces with features at the micrometer scale, comparable in size to bacterial cells, tend to cause cells to align to maximize contact with the surface, whereas surfaces with features much smaller (nanometer scale) than bacterial cells discourage adhesion by reducing the contact area [80].

Different micro and nanostructure patterns have been developed to alter the implant surfaces to enhance their antimicrobial properties [81-84]. Although microscale patterns are not considered bactericidal, they can influence bacterial attachment and biofilm formation. Conversely, the nanoscale patterns can directly damage bacterial cell membranes resulting in bactericidal action. The potential of various nanopatterns, including nanotubes, micropores, nanopores, nanogrooves, and nanopillars, in reducing bacterial attachment is a significant area of research [81-84]. For instance, TiO_2_ nanotubes, created through the anodic oxidation of the Ti surface, exhibited significant resistance to *Porphyromonas gingivalis* (*P. gingivalis*) [85]. The antimicrobial mechanism of those nanotubes involves mutual repulsion of negative charges, stretching of bacterial membranes, and increased surface roughness, preventing the attachment of hydrophobic bacteria [85]. Previous studies also used micropores and nanopores prepared by nitriding and anodic oxidation on the Ti surface to inhibit the adhesion of *Streptococcus mutans* (*S. mutans*) and *P. gingivalis *and* *showed excellent antimicrobial activity [83,86].

Multifunctional Coatings

Many antibiotics, such as gentamicin, amoxicillin, and vancomycin, were utilized in multifunctional coatings on implant surfaces to improve their antimicrobial properties [87]. While previous research has highlighted concerns, including antibiotic resistance, uncontrolled release, short-term antimicrobial efficacy, and potential cytotoxicity, it is essential to note that antibiotics still play a crucial role in managing peri-implant infections [88]. Encouragingly, recent developments in local drug delivery systems, based on promising findings from previous studies, aim to enhance antimicrobial effectiveness, prolong release duration, and mitigate systemic side effects [89]. In light of the global focus on antibiotic resistance, exploring alternative preventive approaches may present a viable solution. 

Polymers have garnered considerable interest because of their natural antimicrobial characteristics and potential application on the implant surface [90-92]. Polysaccharides, the most common macromolecular natural polymer, play a significant role in implantology. Polysaccharide-based coatings improve implant outcomes by preventing infections, promoting tissue integration, and enhancing overall biocompatibility [90-92]. These coatings, mainly consisting of hydrophilic polymers, inhibit the adherence of bacteria and increase osteoblast differentiation and biomineralization. Chitin and its most well-known derivative, chitosan, are natural amino polysaccharide polymers without cytotoxic properties. Chitosan might offer coatings with inherent antimicrobial properties as it has been demonstrated that the interaction between chitosan's cations and anions on the bacterial surface inhibits biosynthetic pathways, hence exerting its antimicrobial activity [90-92]. However, further research and developments are still required to understand the cytotoxicity and coating stability of those polysaccharide coatings.

Polyethylene glycol (PEG) is a polymer frequently used to modify the surface of Ti implants, making the surface hydrophilic and resistant to bacteria [93]. This is due to its flexible chains and strong antifouling properties that help control and prevent the attachment of various bacteria, such as *Staphylococcus epidermidis* (*S. epidermidis*), *Streptococcus sanguinis* (*S. sanguinis*), and *Ligilactobacillus salivarius* (*L. salivarius*), to the implant surface [93]. Combining PEG with specific antibacterial peptide sequences, such as RGD, enhances the attachment of host cells, such as fibroblasts and osteoblasts, while simultaneously preventing bacterial adhesion [93,94]. This approach could improve the performance of biomaterials and medical devices by promoting tissue integration while reducing the risk of infections.

Several metal ions have excellent antimicrobial properties and are also used in coatings for implant surfaces [95,96]. For instance, Ag ions and nanoparticles have an antimicrobial impact on various bacteria associated with peri-implant infection [95]. However, Ag ions have shown low biocompatibility regarding cytotoxicity, one of the main disadvantages of their use in multifunctional coatings [97]. Moreover, using Ag nanoparticles in humans has been linked to both immunotoxic and systemic toxic effects, which raises serious concerns about the impact on general health [98]. These issues could be overcome by combining Ag and another biocompatible metal ion, i.e., Sr, that does not interfere with its release and antimicrobial properties while maintaining a positive impact on osseointegration [99].

Sr is a metal ion that has gained attention for its promising medical therapeutic benefits [100]. In dental implantology, studies have shown that Ti doped with Sr ions enhances bone growth and osseointegration with excellent biocompatible properties [101,102]. Additionally, Sr ions have demonstrated antimicrobial activity against bacteria associated with peri-implantitis, such as *S. aureus*, *E. coli*, and *P. gingivalis* [103,104]. Compared to Ag ions, Sr ions have shown prolonged antimicrobial action against *S. aureus* [105]. Other metallic elements, such as Zn and Cu, have also demonstrated promising antimicrobial properties [29,106].

## Conclusions

Significant research has been conducted on different methods for altering the surface of Ti-based implants. The primary objectives of these methods were to enhance osseointegration by increasing the contact area between the bone and titanium and minimizing bacterial adhesion on the implant surface. This review discussed various implant surface modification methods commonly employed to achieve these objectives. Ti implants are frequently used in dental implantology due to their advantageous mechanical properties but insufficient osseointegration may lead to the formation of fibrous tissue around the implants, primarily because of minimal interaction with the surrounding tissue. Implant surfaces with appropriate roughness and patterns can significantly enhance osseointegration. Moreover, HA coatings on implant surfaces have shown clinical success in promoting osteoconductive ability but these coatings lack bonding strength, possibly causing delamination and implant loosening, requiring the addition of reinforcements. TiO_2_ coatings have emerged as excellent alternatives with improved stability. These two commonly used osteoconductive coatings have been further enhanced by incorporating bioactive elements and antimicrobial agents to improve their function. The selection of a multifunctional approach for surface modification should be based on the desired outcome, the physical and chemical properties of the substrate material, and cost considerations. Furthermore, it is often necessary to use multiple methods to meet the clinical requirements comprehensively. Although surface modification technology offers various advantages and disadvantages, additional methods must be developed and optimized to meet the evolving needs of clinical treatments.
